# Helping people help themselves? Effectiveness of a self-help group for patients with alcohol use disorders—a pilot study

**DOI:** 10.3389/fpsyg.2025.1641718

**Published:** 2025-12-15

**Authors:** Felix Wucherpfennig, Cara Borger, Brian Schwartz

**Affiliations:** 1 Department of Psychology, HSD Hochschule Döpfer University of Applied Sciences, Cologne, Germany; 2 Department of Psychology, Lund University, Lund, Sweden; 3 Department of Psychology, Trier University, Trier, Germany

**Keywords:** group therapy, longitudinal study, symptom distress, self-efficacy, multilevel modeling

## Abstract

**Objective:**

Peer-led self-help groups are increasingly popular, but research on their effectiveness and processes remains scarce. This pilot study aimed to investigate treatment outcome and the impact of Yalom’s therapeutic factors in self-help groups for alcohol use disorders (AUDs).

**Method:**

A self-help (SH, *n* = 32) and a professional-led therapy group (GT, *n* = 19) were assessed over the course of 8 weeks using the General Self-Efficacy Scale (GSE), the Hopkins Symptom Checklist (HSCL-11), and the Therapeutic Factors Inventory (TFI). Multi-level models were applied.

**Results:**

The increase in self-efficacy and therapeutic factors was higher in SH than in GT. The decrease in symptom distress did not differ. All results were adjusted for abstinence and prior experience with group treatment. In SH, moderate to large pre–post effect sizes were found, while GT yielded small effect sizes. Yalom’s therapeutic factors correlated with positive treatment outcomes, but the examination of the cause-and-effect relationship was inconclusive.

**Conclusion:**

This pilot study suggests that the effectiveness of self-help groups is comparable to that of professional-led therapy groups in the accompanying treatment of AUDs. However, the small and unbalanced sample, the open-group design, and the absence of consumption and relapse measures limited inference; larger, multi-site studies with balanced groups are warranted. This pilot study of 51 participants of a self-help and a professional-led therapy group provides preliminary evidence that self-help groups could play an important role in bridging the treatment gap for alcohol use disorders. Yalom’s therapeutic factors, particularly instillation of hope and secure emotional expression, were found to be significantly associated with positive treatment outcome.

## Introduction

Among psychiatric diagnoses, alcohol use disorders (AUDs) have the widest treatment gap worldwide, with 82.7% of affected individuals not receiving treatment (Mekonen et al., 2021). According to the *Substance Abuse and Mental Health Services Administration* (Substance Abuse and Mental Health Services Administration, 2023), the most common reasons for not receiving treatment are that individuals are not ready to stop using, are unable to afford the cost, or do not know where to go for treatment. Additionally, even if affected individuals recognize they need help and can afford therapy, 46% of US psychologists are unable to meet the demand for treatment (American Psychological Association, 2022).

Self-help groups could potentially address several of these issues. They are free of charge, easily accessible, and do not need to be led by a mental health professional (American Psychological Association, 2023). It is no surprise, therefore, that the popularity of self-help groups is rising. There are an estimated 70,000 to 100,000 self-help groups in Germany, and the numbers continue to rise (Heinz, 2022). Wuthnow (1996) estimated that there were over 500,000 self-help groups in the US, totaling 8–10 million members. While we could not find any more recent estimations for the US, it can be assumed that this number will continue to rise as well.

Despite their popularity, there is, however, little scientific evidence as to if and how self-help groups work. Previous studies have addressed their effectiveness for selected conditions and life events (Humphreys et al., 2004; Cacciatore, 2007; Björneklett et al., 2012; Field et al., 2013; Bekkering et al., 2016; Brunelli et al., 2016; Hutchison et al., 2018; Lyons et al., 2021), but process and outcome research efforts are scarce. The present pilot study is a first effort to generate evidence for both outcome effectiveness and factors responsible for change in self-help groups.

According to the *APA Dictionary of Psychology* (2023), self-help groups are described as mostly informal groups composed of individuals who share a common life problem and conduct regular meetings to address it and support each other. They are generally free of charge and the key characteristic is the provision of mutual support among members, which is why they are also referred to as “mutual support” or “peer support” groups. The group leader is typically not a mental health professional, but rather someone who has been or is affected by the same challenges as the other members of the group.

Individual studies have indicated that self-help groups positively influence anxiety after breast cancer treatment (Björneklett et al., 2012), post-traumatic stress after stillbirth (Cacciatore, 2007), pre-natal depression and anxiety (Field et al., 2013), and problem gambling (Hutchison et al., 2018), while meta-analyses by Brunelli et al. (2016) and Lyons et al. (2021) found only trivial to small effects on overall recovery. Peterson et al. (2009) compared wait-list, therapist-led, therapist-assisted, and self-help group interventions for binge-eating disorder. While the therapist-led and -assisted groups had higher abstinence rates at the end of treatment (better short-term outcome), they found no differences at the 6- and 12-month follow-up, suggesting the same long-term outcomes for self-help groups as for therapist-led and -assisted groups. Humphreys (2004) reviewed the effectiveness of self-help groups specific to addiction and concluded that the literature suggested similar effects to those of professional treatment. However, the author also recognizes the shortcomings of existing research (especially the lack of longitudinal studies and comparison groups) and recommends cautious interpretation of the results.

Professional-led group therapy, on the other hand, is an evidence-based treatment for many conditions. In group therapy, one or more psychotherapists provide professional treatment for several patients at the same time (American Psychological Association, 2018). Numerous meta-analyses have shown that professional-led group therapy is effective in the treatment of substance use disorders (Lo Coco et al., 2019) and several other mental disorders (Grenon et al., 2017; Schwartze et al., 2017; Barkowski et al., 2020; Janis et al., 2021). In a meta-analysis of 46 RCTs, Burlingame et al. (2016) investigated individual and group therapy in an identical comparison, in terms of treatments, patients, and dose. They found that the treatments produced equivalent outcomes.

While it is well-established *that* group therapy works, there is still no clear consensus as to *how* it works—i.e., which factors or mechanisms of change are responsible for positive treatment outcome. Research has largely focused on the therapeutic factors by Yalom (1970), which encompass 11 factors of change based on his own experience as a group therapist, the views of other therapists, successfully treated patients, and systematic research (Yalom and Leszcz, 2005). The short-form scales TFI-S (Macnair-Semands et al., 2010) and TFI-19 (Joyce et al., 2011) summarize the 11 factors into four categories: (1) *Instillation of hope*, (2) *secure emotional expression*, (3) *awareness of relational* (or *interpersonal*) *impact*, and (4) *social learning*. According to Macnair-Semands et al. (2010), the categories can be defined as follows: *Instillation of hope* refers to the development of positive expectations of the treatment and the hope for personal change as a result. *Secure emotional expression* involves a sense of safety in the group, allowing members to openly express their emotions during sessions. *Awareness of relational impact* encompasses the insight that one is part of a social system that influences its members and, in turn, is influenced by them. Finally, *social learning* concerns the acquisition of social skills and a resulting change of behavior in social interactions by, for example, watching and imitating others within the group. As part of the validation of the short forms of the Therapeutic Factors Inventory (TFI), their scores were found to be predictive of change in quality of life, psychiatric symptoms, and interpersonal functioning and distress (Macnair-Semands et al., 2010; Joyce et al., 2011). We found only two quantitative studies investigating factors of change in peer-led self-help groups, which all focused on Yalom’s therapeutic factors. Diefenbeck et al. (2014) found that several therapeutic factors emerged in an online support group, and Øygard (2001) found evidence for the association of the therapeutic factors with the outcome of a divorce support group.

In summary, even though the popularity of self-help groups is rising, empirical evidence of their effectiveness is scarce. While individual studies suggest that self-help groups may be a viable alternative to other evidence-based treatment options, such as professional-led group therapy (Peterson et al., 2009), few studies explicitly examine how they compare, especially longitudinally. Furthermore, little is known about the underlying processes and factors of change in self-help groups. Preliminary research points to the relevance of Yalom’s therapeutic factors (Øygard, 2001; Diefenbeck et al., 2014), which have yet to be examined extensively to determine their association with the outcome of group treatments.

The present pilot study aims to investigate the effects of self-help groups for patients with AUDs, and compare these effects to those of a professional-led reference group. The main aim is to examine the following three research questions:

1. Does attending a self-help group contribute to a significant improvement in dealing with AUD, comparable to the effect of professional-led group therapy?
2. Are Yalom’s therapeutic factors significantly correlated with positive treatment outcome in self-help and professional-led group therapy?
3. Do scores in perceived therapeutic factors predict treatment outcome in the following session?

## Method

### Research design

The study was conducted over the course of 8 weeks from March 6th until May 1st, 2023. Process and outcome measurements were taken at eight time points after each therapy session in the self-help group (SH) and professional-led therapy group (GT), respectively. We investigated a naturalistic sample under real-world conditions, meaning the subjects were already or became members of the observed groups during the survey period. Hence, no randomization was carried out. The observed groups were framed as *open-ended* and *open-door*, i.e., there was no fixed start or end point, and members could join or leave the groups at any time. This posed the challenge of high fluctuation, which was met by choosing a continuous, longitudinal design, intention-to-treat analysis (ITT) with multi-level models and nested data. Ethical approval was obtained from the ethics committee of [name of institution]. All participants gave their written informed consent.

### Treatment^1^

Participants in SH participated in weekly sessions provided by *Blaues Kreuz e. V.*, a Christian addiction care association, in West Germany. The care association was secular in orientation and open to everyone regardless of religious affiliation. We examined two parallel groups focusing on AUDs. The treatment capacity was up to 15 participants per group. However, the groups were merged for the analysis because participants switched groups several times during the survey period, as is typical for an open group. The sessions took place once a week and each lasted 90 min. No participant attended sessions more than once a week. The groups were peer-led, though the group leaders only initiated the session before members participated in free-flowing conversations. They primarily focused on sharing experiences, but there were no predefined session topics.

Participants in GT participated in weekly 90-min sessions at a rehabilitation center in West Germany, managed by *Sozialdienst katholischer Männer e. V.* (SKM), a catholic social service association. This treatment was also secular in nature and open to everyone regardless of religious affiliation. Before rehabilitants were admitted to the center, they were required to have completed the withdrawal phase of treatment. At the time of data collection, the participants had stayed at the rehabilitation center for 6 months to 2 years. All participants in this condition were diagnosed with AUDs based on the ICD-10. We examined two weekly therapy groups with a treatment capacity of up to 10 participants per group. The groups were also merged for the data analysis because they were merged twice due to illness during the observation period. The two groups were closely coordinated and discussed the same topics. As opposed to SH, the GT sessions were led by at least two trained addiction therapists per group. To become an addiction therapist in Germany, qualified social workers, social education workers, psychologists, or medical doctors are required to complete 3 years of additional training. The focus of the group was coming to terms with the current situation, coping with the disorder, fostering a supportive environment, and reintegration into work. The professional-led group was chosen as a control group because it was also framed as *open-ended* and *open-door* treatment with fluctuations and heterogeneous previous experiences comparable to the self-help group we examined.

### Participants and assessments

A total of 51 subjects and 200 assessments were examined. No participants completed the assessment at all eight time points. In SH, there were 32 participants. Sixteen participants (50%) completed six assessments, three completed two assessments (9%) and 13 (41%) completed only one assessment. In GT, there were 19 participants. Thirteen of them (69%) completed six assessments, one (5%) completed two assessments, and five (26%) completed only one assessment. Table 1 summarizes the number of assessments for each of the eight time points separately for SH and GT.

**Table 1:** Number of assessments over the study period of 8 weeks.

	SH	GT
	Assessments (cumulative %)	Assessments (cumulative %)
1st session	17 (14.7)	12 (14.1)
2nd session	14 (26.9)	11 (27.0)
3rd session	16 (40.8)	11 (40.0)
4th session	16 (54.7)	10 (51.7)
5th session	15 (67.8)	12 (65.8)
6th session	12 (78.2)	10 (77.6)
7th session	11 (87.8)	11 (90.5)
8th session	14 (100)	10 (100)

SH, self-help group (*n* = 32); GT, professional-led group therapy (*n* = 19). Assessments were collected after each weekly session across eight time points. Analyses followed intention-to-treat with last-observation-carried-forward for post-scores when only one assessment was available and multilevel growth models for longitudinal change.

### Materials

#### Therapeutic factors inventory

For the assessment of the perceived therapeutic factors, we employed the German version of the Therapeutic Factors Inventory–19 (Mander et al., 2016). The TFI-19 is a 19-item measure summarizing Yalom’s 11 therapeutic factors into four categories: *Instillation of hope*, *secure emotional expression*, *awareness of relational impact*, and *social learning*. The items are rated on a 5-point Likert scale, ranging from 0 (strongly disagree) to 4 (strongly agree). The internal consistency of the subscales of the TFI-19 ranges from acceptable to good - *instillation of hope*: α = 0.90; *secure emotional expression*: α = 0.81; *awareness of relational impact*: α = 0.82; *social learning*: α = 0.73 (Mander et al., 2016). In addition to the subscales, the total score of the TFI was used as an average indicator for the perceived therapeutic factors.

#### General self-efficacy scale

We used self-efficacy as our primary outcome measure. The term was coined by Bandura (1977) in his Social Cognitive Theory. He defines self-efficacy as the expectation of personal controllability and competence to achieve a certain outcome. Bandura states that this expectation plays a crucial role in behavioral change by determining whether behavior (in this case, abstinence) is initiated, how much effort will be expended, and how long it will be sustained when being met with obstacles. Several studies suggest that self-efficacy is a predictor of abstinence in substance use disorders (DiClemente et al., 2001; Chavarria et al., 2012; Kelly and Greene, 2013; Müller et al., 2019), and remains stable during follow-up (DiClemente et al., 2001; Müller et al., 2019). We employed the General Self-Efficacy Scale (GSE) (Jerusalem and Schwarzer, 2003), which consists of 10 items that participants rate on a 4-point Likert scale ranging from 0 (not at all true) to 3 (exactly true). It has a high internal consistency in German samples α = 0.80–0.90 (Jerusalem and Schwarzer, 2003).

#### Hopkins symptom checklist

For the secondary outcome measure we employed the German short form of the Symptom Checklist–90 Revised (SCL-90-R), the Hopkins Symptom Checklist (HSCL-11; Lutz et al., 2006). The scale consists of 11 items concerning subjective psychological and physiological symptom distress during the last 7 days. The items are rated on a 5-point Likert scale, ranging from 0 (not at all) to 4 (extremely). Validation assessment suggests high internal consistency and test–retest reliability (*r* = 0.86; (Lutz et al., 2006).

We recorded ‘months abstinent’ at baseline to index stage of recovery and included it as a covariate in all models; abstinence was not analyzed as a primary endpoint. Our outcome focus was self-efficacy and symptom distress (GSE, HSCL-11) and perceived therapeutic processes (TFI).

### Procedure

The groups were assessed using paper-pencil questionnaires after each group session at eight time points. Participants could join the study at any time since there was no fixed start or end point to the treatments. When they first participated in the survey, they were asked to provide informed consent, demographic data, a subjective rating of their prior experience with group sessions, as well as how long they have remained abstinent from alcohol. Finally, they generated an anonymization code to match future responses. At every assessment they were present for, the participants completed the TFI, GSE, and HSCL-11. Each assessment took about 10–15 min.

### Data analyses

We used JASP Version 0.18 (JASP Team, 2023), and R Version 4.3.1 (R Core Team, 2023) to conduct the statistical analyses using an intention-to-treat (ITT) analysis. Missing values in the post-therapy outcome were imputed using last-observation-carried-forward (LOCF). Multi-level models were applied for longitudinal analyses to handle missing values on the session level within an ongoing treatment. This method was chosen because the open setting of the groups naturally produced dropouts that would have diminished the sample size if all subjects with missing data were removed. This means that for subjects who only completed one assessment, the pre- and post-scores were identical.

First, we compared the two groups’ sociodemographic data and initial scores on the GSE, HSCL-11, and TFI using MANOVA, *t*-tests, and chi-squared tests, according to the scale level of the respective variables.

To answer the first research question, we calculated pre-post effect sizes Cohen’s *d* with pooled variances for GSE, HSCL-11, and TFI in SH and GT, respectively. To quantify differences between SH and GT, we calculated Hedges’ *g* with pooled and weighted variances. Additionally, we employed multi-level models to account for non-independence in longitudinal data with sessions nested within patients. In contrast to classical covariance analyses, multi-level models better accommodate differences in the variance–covariance structure and missing values. We calculated three multi-level models with a random intercept and a random slope for the time point for each of the three dependent variables (GSE, HSCL-11, and TFI). In model 1, time point (sessions 1–8) was added as a level-1 (session-level) predictor, and abstinence and experience were added as control variables on level 2. The effect of time point on the respective dependent variable was allowed to vary between patients (random slope). In model 2, group affiliation (SH or GT) was added as a level-2 (patient-level) predictor to the model. In model 3, the interaction between group affiliation and time point was added as a cross-level interaction. For each model, explained variance was calculated as *R^2^,* and ∆*R^2^* was used to identify the change in explained variance due to the main and moderating effects of group affiliation. Additionally, we calculated multi-level models with a random intercept and a random slope for the time point for each of the four subscales of the TFI. Again, time point, group affiliation, and their interaction term were tested as predictors, while the analyses were adjusted for abstinence and experience. For all multi-level models, all continuous predictor variables (i.e., time point, abstinence, and experience) were grand-mean-centered, while group affiliation was recoded to −0.5 (= SH) and 0.5 (= GT).

For the second research question, we calculated three multi-level models for each of the dependent variables, GSE and HSCL-11. In model 1, group affiliation, abstinence, and experience were used as control variables. In model 2, the TFI was added as a fixed effect to test its predictive value for GSE and HSCL-11 in the same session. In model 3, the interaction effect of TFI and group affiliation were added to the models. Additionally, we calculated a multi-level model for each of the four subscales of the TFI, including all control variables (group affiliation, abstinence, and experience) and the interaction term of group affiliation and the respective subscale of the TFI. Since the TFI and the outcome variables (GSE and HSCL-11) were each collected at the same time point, these analyses only examine a correlational relationship.

Finally, we generated lagged variables of GSE, HSCL-11, and TFI to further investigate the relationship between the TFI and outcome in a cause-and-effect model. To answer the third research question, we calculated multi-level models with GSE and HSCL-11 as dependent variables, the lagged TFI and GSE or HSCL-11 variables as fixed effects on level 1, the group affiliation, abstinence, and experience on level 2, and the cross-level interaction between TFI and group affiliation. The lagged TFI variable was used to predict the change in the respective outcome variable (i.e., GSE and HSCL-11) by regressing the outcome at session t on the TFI and the outcome at session t–1.

## Results

### Sample characteristics

A MANOVA showed no significant difference in pre-scores between the two groups (SH and GT; *F*(3, 47) = 1.5, *p* = 0.227, Wilk’s lambda = 0.913). Participants in SH were significantly older (*M* = 51.65; *SD* = 12.48; range 24–72) than the participants in GT (*M* = 36.21; *SD* = 9.69; range 21–58; *t*(28) = 4.6, *p* < 0.001). About 32% (*n* = 10) in SH and 11% (*n* = 2) in GT were female. The groups did not differ significantly in months abstinent (*t*(47) = 1.21, *p* = 0.232), or number of sessions attended before the study (*t*(47) = 1.69, *p* = 0.099). However, the participants in SH reported greater experience (χ^2^(3, *N* = 51) = 23.4, *p* < 0.001). There was no missing demographic data except for two subjects in the SH condition who did not disclose how long they had been abstinent, and how many sessions they had attended before. The sample characteristics are described in detail in Table 2.

**Table 2:** Descriptive data of the sample characteristics.

Sample characteristics	SH (*n* = 32)	GT (*n* = 19)
	*M* (*SD*) or %	*M* (*SD*) or %
Sex (% female)	32.26	10.53
Age *	51.65 (12.48)	36.21 (9.69)
Abstinence (months)	22.23 (49.92)	8.35 (2.04)
Experience * (% high or very high level)	68.75	0.00
Sessions attended before the study (number of sessions)	31.83 (58.65)	8.90 (9.23)

SH, self-help group; GT, professional-led group therapy.

*p < 0.05: significant difference between SH and GT; participants in SH were significantly older and had more experience with therapy than in GT.

*Question 1*: Does attending a self-help group contribute to a significant improvement in dealing with AUD, comparable to the effect of professional-led group therapy?

According to Cohen (1988), effect sizes can be interpreted as small (*d*/*g* = 0.2), moderate (*d*/*g* = 0.5), and large (*d*/*g* = 0.8). Cohen’s *d* indicates descriptively larger pre–post effects in SH than in GT for all variables. For SH, all effect sizes were statistically significant. In SH, there was a moderate effect for symptom distress (HSCL-11; *d* = 0.56, 95% CI [0.18, 0.93]), and large effects for self-efficacy (GSE; *d* = 0.7, 95% CI [0.31, 1.09]) and therapeutic factors (TFI; *d* = 0.87, 95% CI [0.46, 1.28]). For GT, none of the pre–post effect sizes were significant (HSCL-11: *d* = 0.26, 95% CI [−0.21, 0.71]; GSE: *d* = 0.2, 95% CI [−0.26, 0.65]; TFI: *d* = 0.15, 95% CI [−0.31, 0.6]). The differences between both groups were only significant for therapeutic factors (TFI; *g* = 0.68, 95% CI [0.1, 1.27]), indicating a moderate effect size. The group differences were neither significant for symptom distress (HSCL-11; *g* = 0.19, 95% CI [−0.38, 0.76]), nor for self-efficacy (GSE; *g* = 0.31, 95% CI [−0.26, 0.89]).

**Figure 1:**
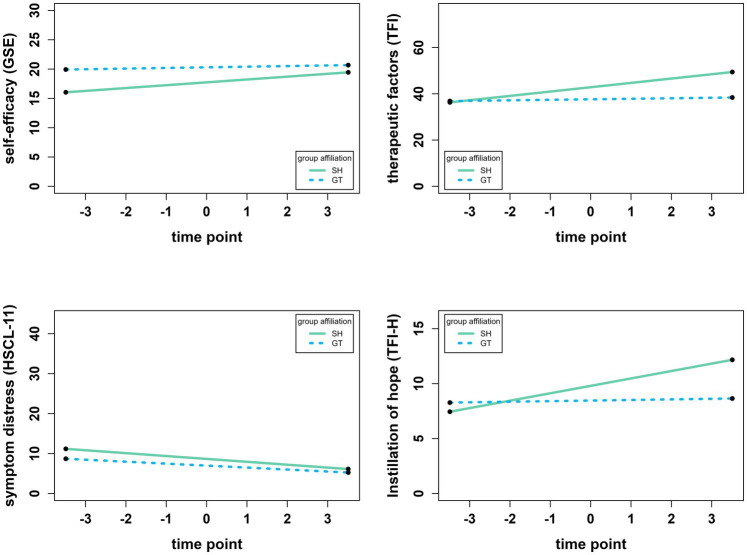
Development of self-efficacy (GSE), therapeutic factors (TFI), symptom distress (HSCL-11), and the subscale “instillation of hope” of the TFI (TFI-H) over time. Analyses used multilevel models with random intercepts and random slopes. Time point is centered around the grand mean of 4.49, i.e., −3.49 refers to session 1 and 3.51 refers to session 8. All y-axes display the whole possible range of the respective instrument. SH = self-help group (*n* = 32), GT = professional-led group therapy (*n* = 19).

The results of all multi-level models regarding question 1 can be found in Supplementary Tables S1–S3. The final analyses showed that the scores for self-efficacy (*b* = 0.297, *p* < 0.001), symptom distress (*b* = −0.607, *p* < 0.001), and therapeutic factors (*b* = 1.047, *p* < 0.001), respectively, changed significantly over time. Self-efficacy and therapeutic factors increased, while symptom distress decreased (cf. Figure 1). For self-efficacy, the interaction of time point and group affiliation (SH vs. GT) was significant (*b* = −0.379, *p* = 0.032) which indicates that despite GT having consistently higher scores (cf. Figure 1), the increase in self-efficacy was higher in SH. The interaction was also significant for therapeutic factors (*b* = −1.652, *p* = 0.005), also indicating a higher increase in SH than in GT. The interaction was not significant for symptom distress (*b* = 0.230, *p* = 0.481), indicating that it decreased equally in both groups. Group affiliation explained between 0.6% (∆*R*^2^ = 0.006 for symptom distress) and 4.2% (∆*R*^2^ = 0.042 for therapeutic factors) in outcome variance beyond time point, abstinence, and experience. The interaction between time point and group affiliation explained another 0.2% (∆*R*^2^ = 0.002 for symptom severity) to 3.7% (∆*R*^2^ = 0.037 for self-efficacy) even beyond the main effect of group affiliation (Supplementary Tables S1–S3).

The main effect of time point was significant for three of the four subscales of the TFI. It was significant for *instillation of hope* (*b* = 0.364, *p* = 0.001), s*ecure emotional expression* (*b* = 0.304, *p* = 0.033), and s*ocial learning* (*b* = 0.189, *p* = 0.004), but not for *awareness of relational impact* (*b* = 0.131, *p* = 219). The interaction effect of time point and group affiliation (SH vs. GT) was significant for *instillation of hope* (*b* = −0.621, *p* = 0.006) and s*ecure emotional expression* (*b* = −0.787, *p* = 0.007), which both increased more steadily in SH. The interaction was not significant for *awareness of relational impact* (*b* = −0.003, *p* = 0.989) and *social learning* (*b* = −0.157, *p* = 0.204), indicating that the score on these subscales increased at the same rate for both groups. Figure 1 visualizes the development of the GSE, HSCL-11, and TFI, as well as the subscale *instillation of hope* (TFI-H) of the TFI as an example.

*Question 2*: Are Yalom’s therapeutic factors significantly related to positive treatment outcome?

The results of all multi-level analyses regarding question 2 can be found in Supplementary Tables S4, S5. The final multi-level models showed significant main effects of the therapeutic factors on both self-efficacy (*b* = 0.089, *p* < 0.001) and symptom distress (*b* = −0.089, *p* = 0.005). The effect was positive for the GSE and negative for the HSCL-11 indicating that stronger perceived therapeutic factors were associated with more self-efficacy and less symptom distress. Therapeutic factors were able to explain 4.7% of GSE’s variance (∆*R*^2^ = 0.047) and 2.0% of HSCL-11’s variance (∆*R*^2^ = 0.020) beyond group affiliation, abstinence, and experience (Supplementary Tables S4, S5). The interaction effects between the therapeutic factors and group affiliation were significant for self-efficacy (*b* = −0.109, *p* = 0.018) and symptom distress (*b* = 0.184, *p* = 0.004) indicating that the effect of the TFI on both GSE and HSCL-11 was more pronounced in the SH group than in GT. The interaction explained another 3.5% (∆*R*^2^ = 0.035 for self-efficacy) to 3.8% (∆*R*^2^ = 0.038 for symptom distress) in outcome variance. When the four subscales of the TFI were considered separately, self-efficacy was significantly associated with *instillation of hope* (*b* = 0.209, *p* = 0.004), *secure emotional expression* (*b* = 0.131, *p* = 0.009), *awareness of relational impact* (*b* = 0.164, *p* = 0.025), and *social learning* (*b* = 0.403, *p* < 0.001). Only *instillation of hope* (*b* = −0.330, *p* < 0.001) and *secure emotional expression* (*b* = −0.136, *p* = 0.045) had significant effects on symptom distress.

*Question 3*: Do scores in perceived therapeutic factors predict treatment outcome measures in the following session?

The results of all multi-level models regarding question 3 can be found in Supplementary Table S6. The multi-level analyses showed no significant effects for the prediction of self-efficacy (*b* = 0.051, *p* = 0.056) or symptom distress (*b* = −0.008, *p* = 0.753) using the total score of the TFI of the previous session. Furthermore, there were no significant interaction effects of TFI with group affiliation (on GSE: *b* = −0.029, *p* = 0.573; on HSCL-11: *b* = 0.033, *p* = 0.501).

## Discussion

Self-help groups are gaining popularity (Heinz, 2022) and could be an important addition to the healthcare system to help bridge the treatment gap (Mekonen et al., 2021), as many psychologists struggle to meet the demand for treatment (American Psychological Association, 2022). However, to date, there is little research on the effectiveness of self-help groups or factors responsible for positive outcomes. For this reason, the present pilot study investigated the effectiveness of peer-led self-help groups for patients with AUDs in comparison to professional-led therapy groups. We examined the relationship between Yalom’s therapeutic factors and treatment outcome in a longitudinal design under real-world conditions. The present study contributes significantly to the understanding of self-help groups, as it is one of the first studies to implement an active reference group design with a focus on process and outcome research.

### Sample characteristics

It is of note that our sample consists of more males than females in both conditions. This is in line with the finding that more men than women are diagnosed with AUDs (Substance Abuse and Mental Health Services Administration, 2023). The mean age and previous experience with group therapy also differed significantly between SH and GT. This could be explained by the different treatment stages associated with GT and SH. Group therapy usually takes place during the inpatient or residential rehabilitation phase, while self-help groups are generally recommended for the aftercare phase. Therefore, participants in SH have commonly completed a larger part of the treatment process already. This is corroborated by older, more experienced patients in SH, with a longer duration of alcohol abstinence and more sessions previously attended than in GT. Systematic differences in other variables, like quality of treatment, cannot be ruled out. This issue will be addressed in more detail in the limitations section.

*Question 1*: Does attending a self-help group contribute to a significant improvement in dealing with AUD, comparable to the effect of professional-led group therapy?

In line with previous findings our results generally suggest that self-help groups are effective in the treatment of AUDs and treatment outcome is comparable to that of professional-led group therapy (Humphreys et al., 2004; Bekkering et al., 2016). We also found evidence that self-help groups increase self-efficacy more effectively than professional-led group therapy. While Hedges’ *g* indicated no significant differences between SH and GT for the outcome measures (self-efficacy and symptom distress), Cohen’s *d* indicated a moderate effect for symptom distress and a large effect for self-efficacy in SH. For GT, the effect sizes were small and not significant. The lack of significance is likely related to the smaller sample size in conjunction with the high fluctuation and the unequally distributed sample size between SH and GT. The results are therefore preliminary and should be interpreted cautiously.

The greater experience and longer duration of abstinence in SH alone cannot adequately explain the differences found between SH and GT, as all analyses controlled for these variables. Our findings are especially compelling, considering that Fiorentine (1999) found that only weekly or more frequent participation in 12-step programs was associated with abstinence, while less than weekly participation was not. In our sample, only half of the subjects participated in the groups at six time points, while no one participated every week during our eight-week survey period. Our intention-to-treat and last-observation-carried-forward analysis thus leads to a conservative, but practice-oriented assessment of the effectiveness of the treatment. It is therefore probable that if patients participate more frequently in self-help group sessions than the participants in our sample did, self-help groups might prove even more effective.

#### Self-efficacy

The multi-level model indicated that self-efficacy increased more rapidly in SH than in GT. This suggests that self-help groups might be superior to group therapy in building self-efficacy. A study by Hutchison et al. (2018) indicates that actively providing social support to others increases self-efficacy. Self-help groups likely provide more opportunities to do so, due to more free-flowing discussions and participant interactions (Abu Hassan Shaari and Waller, 2023; Nalbantoğlu and Tuncay, 2024). It is, however, also possible that there was simply less room to grow for the participants in GT because their self-efficacy scores were consistently higher to begin with. Our findings differ from those of previous studies. Two studies included in the review by Lyons et al. (2021) reported findings on self-efficacy in self-help groups. Both focused on peer-led anti-stigma interventions, but neither of them found evidence of an effect on self-efficacy (Rüsch et al., 2014; Russinova et al., 2014). Unfortunately, we could not find any studies that examined self-efficacy in self-help groups for AUDs. Further research is therefore needed to verify our findings.

#### Symptom distress

The decrease in symptom distress did not differ between SH and GT, as indicated by multi-level modeling. This is in line with the meta-analysis by Lo Coco et al. (2019) that indicated no significant differences in the improvement of substance use disorder symptoms between group therapy and other active treatments. Additionally, a study by Bright et al. (1999) indicated that groups led by professionals vs. non-professionals were equally effective in improving depressive symptoms. Our findings provide evidence for a similar effect concerning symptoms related to AUDs.

#### Therapeutic factors

Multi-level models suggested a steadier increase of the TFI in SH than in GT, which is also indicated by a moderate Hedges’ *g*. SH yielded large Cohen’s *d* effect sizes, whereas in GT there were only small, non-significant effects. Given the emphasis on participant interaction in free-flowing discussions in SH, it might have been easier to build relationships with fellow participants, thereby leading to a more rapid increase of the therapeutic factors in comparison to GT, wherein the therapist assumed a more active role.

Considering the subscales of the TFI separately, *instillation of hope* differed the most between SH and GT. Yalom and Leszcz (2005) state that self-help groups typically place special emphasis on this factor by consisting largely of testimonials and members sharing their hardships but eventual recovery. This is seemingly reflected in our findings. Another factor that differed substantially between the two groups was s*ecure emotional expression*. This factor refers to a sense of safety within the group, and comfortably and openly expressing one’s emotions (Macnair-Semands et al., 2010). Again, the emphasis on sharing experiences while fellow members provide support and insight in self-help groups could explain the higher scores in SH. In addition, the participants in SH had attended more sessions prior to the study than those in GT and had therefore potentially already built a stronger bond with each other. Generally, our findings are in line with the theoretical conceptualization of the therapeutic factors.

*Question 2*: Are Yalom’s therapeutic factors significantly related to positive treatment outcome?

Our results indicate that Yalom’s therapeutic factors are significantly associated with the improvement of both self-efficacy and symptom distress. When considering the subscales of the TFI, all of them are significantly related to self-efficacy. Self-efficacy, in this context, refers to the belief that one can recover from one’s addiction. It is conceivable that other members demonstrating that recovery is possible (*instillation of hope*), and sharing the skills and tools that helped them (*social learning*), as well as being part of a supportive social system (*awareness of relational impact*) in which one feels safe (*secure emotional expression*) could influence that belief. However, reverse causality is also feasible: Higher self-efficacy could lead to patients believing that recovery is possible (*instillation of hope*) and therefore, they become more willing to learn new skills that could help them (*social learning*). Additionally, self-efficacy might influence the belief in one’s meaningful impact on others (*awareness of relational impact*) and being comfortable sharing one’s emotions (*secure emotional expression*).

For symptom distress, only *instillation of hope* and *secure emotional expression* had a significant effect. Again, the direction of causality is unclear. One possibility is that the more patients believe in recovery (*instillation of hope*) and the safer they feel in expressing their emotions (*secure emotional expression*), the less challenging the symptoms might become. Another explanation—and perhaps the more plausible of the two—is that the lower the patients’ symptom distress, the higher their belief in a possible recovery (*instillation of hope*) in addition to less challenging emotions which, in turn, are easier to share with the group (*secure emotional expression*).

Our findings are in line with previous studies that indicated an association between Yalom’s therapeutic factors and positive treatment outcome in other types of treatment (Øygard, 2001; Macnair-Semands et al., 2010; Joyce et al., 2011). The present study provides support for the factors’ applicability to self-help groups with a focus on AUDs. We found evidence that the subscales *instillation of hope* and *secure emotional expression* of the TFI are of particular interest regarding the effectiveness of group treatment. Future studies should investigate this relationship further to replicate our findings.

*Question 3*: Do scores in perceived therapeutic factors predict treatment outcome measures in the following session?

We could not predict treatment outcome measures in the following session through Yalom’s therapeutic factors. It is possible that our sample size was simply too small to determine any effects, which could be indicated by the effect of therapeutic factors on self-efficacy in the following session showing borderline significance (*p* = 0.056). However, it might also suggest that the therapeutic factors are at least not the only factors responsible for the effectiveness of group treatments and might interact with other factors. It is also possible that the cause-and-effect relationship is reversed: That is, the therapeutic factors might not lead to recovery, but recovery might increase the therapeutic factors, as outlined in the previous section. A study by Dinger et al. (2020) similarly suggests that group cohesion is not only a predictor but also an outcome of symptom reduction. As group cohesion is also one of Yalom’s original therapeutic factors, these findings might be related. As our findings are non-conclusive, further studies are needed to determine the existence and direction of the cause-and-effect relationship and establish whether nurturing specific factors could increase the effectiveness of group treatments.

## Limitations and recommendations

It is important to emphasize the naturalistic design of the present study. While RCTs can provide more reliable results and are the gold standard for investigating cause-and-effect relationships, our primary goal was not to conduct rigorous between-group comparisons, but rather an initial evaluation of self-help groups under real-world conditions, in which the professional-led therapy group served as a reference rather than a true control group. Nevertheless, RCTs on the effectiveness of self-help groups should be implemented in the future The RCT study design should plan for balanced samples via multi-site recruitment, conduct *a priori* power analyses for multilevel growth models, pre-register hypotheses, and may apply longer observation windows and follow-up assessments to support stronger inferences.

It is also important to note that we were only able to investigate one self-help group and one professional-led therapy group. Systematic differences regarding, for example, the quality of the treatments, content, group roles, member personalities, or leader behavior cannot be ruled out. Further studies should be conducted to examine whether our results can be generalized to different groups and group compositions.

Furthermore, the high fluctuation in our sample—e.g., 41% of the participants in SH participated in only one session—is of particular note, though the reasons are unclear. We met these fluctuations by employing the last-observation-carried-forward method (LOCF) which leads to more conservative estimations of the effect sizes and multi-level models by having identical pre- and post-treatment scores for those who only participated once.

Another limitation of this study is its small sample size. While promising effects emerged, it is advisable to replicate the study with a larger sample size, as our sample was likely too small and unbalanced to reliably show all effects. Especially the non-significant effect sizes in GT contradict the numerous meta-analyses that have shown that professional-led group therapy is a highly effective treatment option for many conditions (Schwartze et al., 2016; Grenon et al., 2017; Barkowski et al., 2020; Janis et al., 2021), including substance use disorders (Lo Coco et al., 2019), and leads to equivalent outcomes as individual therapy (Burlingame et al., 2016). It is therefore probable that the non-significant effect sizes we found are not reflective of the ineffectiveness of GT, but rather constitute a limitation of our study. The effect sizes Cohen’s *d* we estimated for GT should therefore be interpreted cautiously, as should Hedges’ *g* as a comparison of the conditions. However, as the effect sizes for SH were significant, they likely represent the actual effectiveness of the intervention and provide evidence for the positive effect of self-help groups. In the future, RCTs should be conducted to provide more reliable evidence of the effectiveness of self-help groups.

While we found a significant association of Yalom’s therapeutic factors with positive treatment outcome, we could not establish the direction of the cause-and-effect relationship. Future studies should examine whether a longer assessment period leads to more conclusive results. It is also possible that additional factors might be relevant for the effectiveness of group treatments. For example, Kelly and Greene (2013) and Müller et al. (2019) found evidence for the role of an interplay between motivation for recovery and self-efficacy in predicting the outcome of residential treatment for substance use disorder. Furthermore, a study on problem gambling by Hutchison et al. (2018) suggests that the *provision* of social support (rather than its *receipt*) in self-help groups plays a crucial role in increasing self-efficacy and decreasing the risk of (re)lapse in trigger situations. If future studies find that the provision of social support is higher in self-help groups, this could also explain the greater increase in self-efficacy we found in SH. Therefore, Yalom’s therapeutic factors, as well as motivation for sobriety and provision of social support should be further investigated.

We chose to use general outcome measures rather than specific measures for abstinence or dependence. Alcohol addiction is often comorbid with other disorders (Castillo-Carniglia et al., 2019), which is why the assessment of symptom distress and the patients’ well-being is of importance. Furthermore, self-efficacy represents a relevant construct for the maintenance of alcohol abstinence (Chavarria et al., 2012). This indirect survey of abstinence may reduce the social desirability bias and the stigmatization of respondents. However, since we did not measure changes in consumption (quantity, frequency) and relapse events, changes in addictive behavior per se could not be evaluated. Unobserved variation in use patterns may have influenced outcomes. Future studies should consider both direct and indirect assessments of alcohol addiction, as they might have different implications.

Although our outcomes (self-efficacy, symptom distress, therapeutic factors) were not contingent on abstinence, both study settings (SH; GT) encouraged abstinence as a treatment goal. Individuals who did not wish to stop using might have faced program-fit barriers (e.g., perceived moral incongruence, fear of stigma, concerns about mandated abstinence). Self-help groups might nonetheless reduce harm by providing non-judgmental social support, modeling safer-use strategies, and enabling incremental goal setting (e.g., delaying first drink, reducing heavy-drinking days). We did not assess constructs related to pleasure from alcohol use or goal-directed management of use; therefore, our findings cannot adjudicate benefits for participants pursuing non-abstinence goals. Future studies may include consumption-level indices, goal-attainment scaling, and measures of drinking motives/pleasure to test whether peer-led groups facilitate harm reduction pathways as well as abstinence.

All data were collected in Western Germany within Christian-affiliated providers; therefore, cultural and system-level factors may limit generalizability. In the German healthcare system, it is not uncommon for psychotherapeutic treatments to be provided by Christian organizations. These are generally secular in orientation and open to people of all faiths. Nevertheless, it could not be ruled out that religious framing might have shaped group norms (e.g., emphasis on abstinence, moral language use) and leadership styles. Although attendance was not restricted by belief and we did not measure religiosity, these contextual features could have influenced who engaged, which mechanisms were activated (e.g., hope, belonging), and how transferable the effects were to secular settings. For this reason, it could be relevant to assess religiosity in future studies.

Lastly, there is still a gap in research regarding the differential effectiveness of self-help groups for different conditions, symptom severities, consistency of participation, additional support or aids, comorbid diagnoses, and previous treatment. Comparative studies are therefore needed to establish clear recommendations.

## Conclusion

In summary, the present study suggests that the effectiveness of self-help groups is comparable to that of professional-led therapy groups for patients with AUDs. Additionally, we found evidence that self-help groups might be more effective in increasing self-efficacy, supposedly due to their emphasis on the provision of mutual support. Yalom’s therapeutic factors were related to positive treatment outcome, though the direction of the cause-and-effect relationship, as well as the influence of other potential factors, remains unclear. Further research with larger sample sizes and RCT designs is needed to better understand the relationship between process and outcome in group treatments. Insights gained from research in this area could help therapists, group leaders, and participants make better research-informed decisions. Still, our findings imply that self-help groups could play a valuable role in bridging the treatment gap for AUD patients and should be the subject of further research.

## Data Availability

The raw data supporting the conclusions of this article will be made available by the authors, without undue reservation.

## Footnotes

1 Harm reduction and abstinence represent complementary approaches within the continuum of care for alcohol use disorders. Harm reduction emphasizes engagement without requiring abstinence and prioritizes incremental risk reduction (e.g., managed-use goals, medication-assisted treatment, safer-use education), whereas abstinence-oriented programs explicitly aim at sustained non-use. The GT setting and to a lesser extent the SH setting were embedded in abstinence-oriented service structures; therefore, our findings pertain to abstinence-oriented contexts and may not generalize to programs whose primary aim is managed use.
