# Supplementary material for: Helping people help themselves? Effectiveness of a self-help group for patients with alcohol use disorders—a pilot study
Source: Front Psychol. 2025 Dec 15;16:1641718. doi: 10.3389/fpsyg.2025.1641718 (PMC12745289; doi:10.3389/fpsyg.2025.1641718)
Supplement: Supplementary file 1 [file Table_1.DOCX]

**Supplemental Material**

**Table S1**

*Multi-level models predicting self-efficacy measured with the GSE in self-help groups and group therapy*

|  | Model 1 | | Model 2 | | Model 3 | |
| --- | --- | --- | --- | --- | --- | --- |
|  | Estimate | p-value | Estimate | p-value | Estimate | p-value |
| Fixed effects |  |  |  |  |  |  |
| Intercept | **18.799** | **< .001** | **18.948** | **< .001** | **19.018** | **< .001** |
| Time point | **0.323** | **< .001** | **0.322** | **< .001** | **0.297** | **< .001** |
| Abstinence | 0.024 | .139 | 0.026 | .123 | 0.024 | .146 |
| Experience | **1.746** | **.012** | 1.298 | .135 | 1.270 | .139 |
| Group affiliation |  |  | 1.748 | .337 | 2.563 | .165 |
| Time point*group  affiliation |  |  |  |  | **–0.379** | **.032** |
| Random effects |  |  |  |  |  |  |
| Residual (level 1) | 4.465 |  | 4.483 |  | 4.400 |  |
| Intercept (level 2) | 22.204 |  | 22.041 |  | 21.568 |  |
| Time point (level 2) | 0.039 |  | 0.029 |  | 0.029 |  |
| R² marginal | .122 |  | .144 |  | .181 |  |
| R² conditional | .854 |  | .856 |  | .862 |  |

*Note.* GSE = General Self-Efficacy Scale. Significant effects are highlighted in bold. R² marginal indicates variance explained by all fixed effects. R² conditional indicates variance explained by all fixed and random effects.

**Table S2**

*Multi-level models predicting symptom distress measured with the HSCL-11 in self-help groups and group therapy*

|  | Model 1 | | Model 2 | | Model 3 | |
| --- | --- | --- | --- | --- | --- | --- |
|  | Estimate | p-value | Estimate | p-value | Estimate | p-value |
| Fixed effects |  |  |  |  |  |  |
| Intercept | **7.935** | **< .001** | **7.820** | **< .001** | **7.817** | **< .001** |
| Time point | **–0.618** | **< .001** | **–0.620** | **< .001** | **–0.607** | **< .001** |
| Abstinence | –0.023 | .303 | –0.024 | .288 | –0.024 | .306 |
| Experience | –1.028 | .251 | –0.552 | .628 | –0508 | .658 |
| Group affiliation |  |  | –1.507 | .532 | –1.675 | .491 |
| Time point*group  affiliation |  |  |  |  | 0.230 | .481 |
| Random effects |  |  |  |  |  |  |
| Residual (level 1) | 6.090 |  | 6.087 |  | 6.091 |  |
| Intercept (level 2) | 35.427 |  | 36.206 |  | 36.729 |  |
| Time point (level 2) | 0.532 |  | 0.530 |  | 0.528 |  |
| R² marginal | .070 |  | .076 |  | .078 |  |
| R² conditional | .871 |  | .874 |  | .876 |  |

*Note.* HSCL-11 = Hopkins Symptom Checklist-11. Significant effects are highlighted in bold. R² marginal indicates variance explained by all fixed effects. R² conditional indicates variance explained by all fixed and random effects.

**Table S3**

*Multi-level models predicting therapeutic factors measured with the TFI in self-help groups and group therapy*

|  | Model 1 | | Model 2 | | Model 3 | |
| --- | --- | --- | --- | --- | --- | --- |
|  | Estimate | p-value | Estimate | p-value | Estimate | p-value |
| Fixed effects |  |  |  |  |  |  |
| Intercept | **40.511** | **< .001** | **39.932** | **< .001** | **40.216** | **< .001** |
| Time point | **1.119** | **< .001** | **1.133** | **< .001** | **1.047** | **< .001** |
| Abstinence | –0.041 | .274 | –0.045 | .220 | –0.050 | .193 |
| Experience | –1.677 | .277 | 0.279 | .884 | 0.111 | .955 |
| Group affiliation |  |  | –7.074 | .083 | –5.215 | .217 |
| Time point*group affiliation |  |  |  |  | **–1.652** | **.005** |
| Random effects |  |  |  |  |  |  |
| Residual (level 1) | 45.788 |  | 45.517 |  | 45.438 |  |
| Intercept (level 2) | 99.508 |  | 97.405 |  | 103.491 |  |
| Time point (level 2) | 0.949 |  | 0.988 |  | 0.143 |  |
| R² marginal | .067 |  | .109 |  | .106 |  |
| R² conditional | .714 |  | .724 |  | .728 |  |

*Note.* TFI = Therapeutic Factors Inventory. Significant effects are highlighted in bold. R² marginal indicates variance explained by all fixed effects. R² conditional indicates variance explained by all fixed and random effects.

**Table S4**

*Multi-level models predicting self-efficacy measured with the GSE in self-help groups and group therapy by therapeutic factors measured with the TFI*

|  | Model 1 | | Model 2 | | Model 3 | |
| --- | --- | --- | --- | --- | --- | --- |
|  | Estimate | p-value | Estimate | p-value | Estimate | p-value |
| Fixed effects |  |  |  |  |  |  |
| Intercept | **18.979** | **< .001** | **18.956** | **< .001** | **18.842** | **< .001** |
| Group affiliation | 2.367 | .213 | 2.791 | .135 | 2.903 | .114 |
| Abstinence | 0.030 | .103 | .034 | .057 | **0.038** | **.033** |
| Experience | 1.563 | .084 | 1.553 | .079 | 1.589 | .067 |
| TFI |  |  | **.078** | **< .001** | **0.089** | **< .001** |
| TFI*group affiliation |  |  |  |  | **–0.109** | **.018** |
| Random effects |  |  |  |  |  |  |
| Residual (level 1) | 5.029 |  | 4.753 |  | 4.657 |  |
| Intercept (level 2) | 21.912 |  | 20.861 |  | 20.075 |  |
| R² marginal | .190 |  | .237 |  | .272 |  |
| R² conditional | .849 |  | .858 |  | .863 |  |

*Note.* GSE = General Self-Efficacy Scale; TFI = Therapeutic Factors Inventory. Significant effects are highlighted in bold. R² marginal indicates variance explained by all fixed effects. R² conditional indicates variance explained by all fixed and random effects.

**Table S5**

*Multi-level models predicting symptom distress measured with the HSCL-11 in self-help groups and group therapy by therapeutic factors measured with the TFI*

|  | Model 1 | | Model 2 | | Model 3 | |
| --- | --- | --- | --- | --- | --- | --- |
|  | Estimate | p-value | Estimate | p-value | Estimate | p-value |
| Fixed effects |  |  |  |  |  |  |
| Intercept | **7.906** | **< .001** | **7.936** | **< .001** | **8.145** | **< .001** |
| Group affiliation | –1.524 | .522 | –1.909 | .430 | –2.120 | .393 |
| Abstinence | –0.029 | .208 | –0.033 | .159 | –0.039 | .102 |
| Experience | –0.649 | .563 | –0.638 | .574 | –0.700 | .549 |
| TFI |  |  | **–0.070** | **.027** | **–0.089** | **.005** |
| TFI*group affiliation |  |  |  |  | **0.184** | **.004** |
| Random effects |  |  |  |  |  |  |
| Residual (level 1) | 9.767 |  | 9.443 |  | 8.851 |  |
| Intercept (level 2) | 34.164 |  | 35.277 |  | 37.578 |  |
| R² marginal | .055 |  | .075 |  | .113 |  |
| R² conditional | .790 |  | .805 |  | .831 |  |

*Note.* HSCL-11 = Hopkins Symptom Checklist-11; TFI = Therapeutic Factors Inventory. Significant effects are highlighted in bold. R² marginal indicates variance explained by all fixed effects. R² conditional indicates variance explained by all fixed and random effects.

**Table S6**

*Multi-level models predicting GSE and HSCL-11 at time point T1 by therapeutic factors measured with the TFI at T0*

|  | GSE model | | HSCL-11 model | |
| --- | --- | --- | --- | --- |
|  | Estimate | p-value | Estimate | p-value |
| Fixed effects |  |  |  |  |
| Intercept | **18.857** | **< .001** | **6.077** | **< .001** |
| Outcome lagged^1^ | **0.209** | **.012** | **0.599** | **< .001** |
| Group affiliation | 0.821 | .507 | –0.467 | .559 |
| Abstinence | **0.030** | **.016** | –0.005 | .506 |
| Experience | **1.739** | **.016** | 0.294 | .475 |
| TFI lagged | 0.051 | .056 | –0.008 | .753 |
| TFI lagged*group  affiliation | –0.029 | .573 | 0.033 | .501 |
| Random effects |  |  |  |  |
| Residual (level 1) | 4.829 |  | 7.507 |  |
| Intercept (level 2) | 5.701 |  | 1.138 |  |
| R² marginal | .464 |  | .487 |  |
| R² conditional | .754 |  | .555 |  |

*Note.* GSE = General Self-Efficacy Scale; HSCL-11 = Hopkins Symptom Checklist-11; TFI = Therapeutic Factors Inventory. Significant effects are highlighted in bold. R² marginal indicates variance explained by all fixed effects. R² conditional indicates variance explained by all fixed and random effects.

^1^outcome refers to the respective instrument used as the prediction criterion, i.e., GSE or HSCL-11
